# Phase I clinical trials of safety and immunogenicity of live cultural influenza vaccine vector-flu

**DOI:** 10.1186/1753-6561-9-S9-P73

**Published:** 2015-12-14

**Authors:** Elena A Nechaeva, Marina P Bogryantseva, Alexander B Ryzhikov, Ol'ga G P'yankova, Tatyana Y Sen'kina, Irina F Radaeva, Sergey Y Nechaev, Natalya I Ryndyuk, Vladimir I Kuzubov, Zoya I Gin'ko, Irina V Kiseleva, Larisa G Rudenko

**Affiliations:** 1 Department of Cell Culture Technology, SRC VB VECTOR, Novosibirsk, 630559, Russia; 2 SRC VB VECTOR, Novosibirsk, 630559, Russia; 3 FSHO Medical Unit #163 FMBA, Koltsovo, Novosibirsk Region, 630559, Russia; 4 Institute of Experimental Medicine, Russian Academy of Medical Sciences, St. Petersburg, 197376, Russia

## Background

Live influenza vaccines trigger all major components of the anti-flu immune response machinery and have been included in the global WHO program on the pandemic preparedness. Key advantages of using live vaccines include a feasibility of intranasal administration, opportunity to rapidly scale up production of the viral substance, simplicity of vaccination, and robust protection against antigenic drift variants of the virus. Use of certified cell cultures for the cultivation of seasonal strains of influenza along with biodegradable materials for constructing delivery vehicles is considered one of the mainstream approaches to the development of new generations of flu vaccines.

We developed a live cultural influenza vaccine called Vector-Flu, which is based on the cold-adapted virus strain A/17/California/2009/38 (H1N1) and MDCK cell line obtained from the certified cell culture depository. Preclinical studies have demonstrated safety and high immunogenicity of Vector-Flu in a ferret model.

Phase I of clinical trials was conducted on healthy volunteers in the Medical Unit #163 in Koltsovo, Russia. The trial pursued the following goals:1) Evaluation of safety and tolerability.2) Evaluation of the humoral and adaptive immune response using HI, ELISA and microneutralization assay.3) Evaluation of the cellular immune response, as measured by the cytokine release level in response to the ex vivo stimulation of blood lymphocytes by the influenza virus.

## Materials and methods

### Cell culture

MDCK from Cell Culture Collection of SRC VB VECTOR. Cells were passed in serum-free SFM4MegaVir medium (USA). The characteristics of the MDCK cell line were studied in accordance with WHO [1].

### Viruses

The vaccine strain A/17/California/2009/38(H1N1) was generated at the Institute of Experimental Medicine (St. Petersburg, Russia) by reassortment of the cold-adapted attenuated A/Leningrad/134/17/57 (H2N2) master donor virus with the pandemic strain A/California/7/2009 (H1N1). The A/Chita/3/2009(H1N1) influenza virus was obtained from VECTOR's Collection of Microorganisms.

### Determination of influenza virus infectious activity

The infectious activity of influenza virus was determined by titration in 10-12-day-old chick embryos. 10-fold dilutions (0.2 ml) of virus-containing fluid were inoculated into the allantoic cavity of chick embryos. The embryos were incubated for 48 hours at a temperature of 35°C. After the incubation, the allantoic fluid was harvested from the embryos to determine the virus infectious activity by agglutination reaction with 1% chicken red blood cells. The virus titer was calculated according to the Reed-Muench method and expressed as log EID50/0.2 ml.

### Control of immunogenicity of the vector-flu vaccine

Hemagglutination Inhibition (HI) test. HAI was performed by a routine technique [2] with some modifications. The assayed sera were pre-treated with the receptor destroying enzyme (RDE). The hemagglutination reaction was performed with 1% chicken red blood cells (RBC). The HAI titer was determined as the reciprocal dilution of the last row which contained non-agglutinated RBC.

Microneutralization assay. The assay was performed in compliance with the WHO guidelines [2] with some modifications. MDCK cells supplemented with equal volumes of serum and influenza virus were mixed and incubated in 5% CO2 at 37°C. The presence of the virus was detected by enzyme immunoassay using the monoclonal antibodies to type A influenza virus NP protein (CDC, Atlanta). Neutralizing antibody titer was defined as reciprocal of the highest serum dilution that provided 50% inhibition of the virus growth in cell culture.

Phase I of clinical trials included 3 arms:

Arm 1 (n = 20): a treatment group. Volunteers were vaccinated using a single dose of the Vector-Flu vaccine containing 106 EID50 of the influenza virus.

Arm 2 (n = 20): a treatment group. Volunteers were vaccinated twice over a course of 10 days using Vector-Flu vaccine containing 106 EID50 of the influenza virus.

Arm 3 (n = 20): a placebo control group. Volunteers were injected twice over a course of 10 days using sterile sodium chloride.

## Results

Our findings show that the Vector-Flu has high tolerability and no significant side effects. Dynamic changes of the hematologic analysis values and urine test results obtained after the immunization were within a normal range. Virus was not detectable in nasal mucus and blood sera of the healthy volunteers after a single and double injections as early as at day 1, demonstrating a rapid clearance of the live virus from the vaccination sites. Additionally, no indications of infection generalizations were observed.

A seroconversion level (number of subjects with >4x increase of the antibody titer) was detected at 45% after a single injection, as measured using HI assay. After the second injection, peaks of immunogenic activity were recorded at 2 and 3 weeks, and seroconversion level rose up to 80%.

Activation of the Th2 immune response was measured in whole blood cells. Post-vaccination cytokine indexes were high and stable: 100% for IL-10 and TNFα, and 75% for IL-6.

## Conclusions

Our findings show that the Vector-Flu has high tolerability and no significant side effects. Clinical trials of the live cultural anti-influenza vaccine Vector-Flu have been conducted in accordance with the protocol of clinical trials and national and international clinical trial guidelines (ICH GCP). The Ministry of Health of Russia has granted a permission to advance to the phase II trials for the Vector-Flu vaccine.
